# Effect of surface treatment strategies on bond strength of additively and subtractively manufactured hybrid materials for permanent crowns

**DOI:** 10.1007/s00784-024-05767-3

**Published:** 2024-06-13

**Authors:** Zhen Mao, Franziska Schmidt, Florian Beuer, Jamila Yassine, Jeremias Hey, Elisabeth Prause

**Affiliations:** 1 Department of Prosthodontics, Geriatric Dentistry and Craniomandibular Disorders, Charité- Universitätsmedizin Berlin, corporate member of Freie Universität Berlin and Humboldt-Universität zu Berlin, Aßmannshauser Str. 4-6, 14197 Berlin, Germany; 2 Department of Prosthodontics, School of Dental Medicine, Martin-Luther-University, Magdeburger Str. 16, 06112 Halle, Germany

**Keywords:** Additive manufacturing, 3D printing, Shear bond strength, Surface roughness, Surface energy

## Abstract

**Objectives:**

The purpose of this study is to evaluate the bond strength of different computer-aided design / computer-aided manufacturing (CAD/CAM) hybrid ceramic materials following different pretreatments.

**Methods:**

A total of 306 CAD/CAM hybrid material specimens were manufactured, *n* = 102 for each material (VarseoSmile Crown^plus^ [VSCP] by 3D-printing; Vita Enamic [VE] and Grandio Blocs [GB] by milling). Each material was randomly divided into six groups regarding different pretreatment strategies: control, silane, sandblasting (50 μm aluminum oxide particles), sandblasting + silane, etching (9% hydrofluorics acid), etching + silane. Subsequently, surface roughness (Ra) values, surface free energy (SFE) were measured. Each specimen was bonded with a dual-cured adhesive composite. Half of the specimens were subjected to thermocycling (5000 cycles, 5–55 °C). The shear bond strength (SBS) test was performed. Data were analyzed by using a two-way analysis of variance, independent t-test, and *Mann-Whitney-U-test* (α = 0.05).

**Results:**

Material type (*p* = 0.001), pretreatment strategy (*p* < 0.001), and the interaction (*p* < 0.001) all had significant effects on Ra value. However, only etching on VSCP and VE surface increased SFE value significantly. Regarding SBS value, no significant difference was found among the three materials (*p* = 0.937), while the pretreatment strategy significantly influenced SBS (*p* < 0.05). Etching on VSCP specimens showed the lowest mean value among all groups, while sandblasting and silane result in higher SBS for all test materials.

**Conclusions:**

The bond strength of CAD/CAM hybrid ceramic materials for milling and 3D-printing was comparable. Sandblasting and silane coupling were suitable for both millable and printable materials, while hydrofluoric etching should not be recommended for CAD/CAM hybrid ceramic materials.

**Clinical relevance:**

Since comparable evidence between 3D-printable and millable CAD/CAM dental hybrid materials is scarce, the present study gives clear guidance for pretreatment planning on different materials.

## Introduction

Due to continuous developments computer-aided design/computer-aided manufacturing (CAD/CAM) has become an integral part of digital dentistry [1–3]. The dominant production method is subtractive manufacturing (milling), which is commonly used for permanent restorations including metals, ceramics, and resin composites [4]. Studies showed that these materials tended to be less fragile than conventional ceramics [5, 6], leading to less fractures at the margins [5–7]. In addition to milling, additive manufacturing (3D-printing) is increasingly coming to the fore. Additive manufacturing refers to the step-by-step and layer-by-layer manufacturing of a three-dimensional (3D) restoration [1, 2]. Compared to subtractive manufacturing, *3D-printing* is more economical in material consumption, offers affordable material costs, and a faster digital workflow. However, due to the requirements of flowable consistency during 3D-printing, hybrid ceramic resins usually include less inorganic fillers compared to millable hybrid ceramic materials. Thus, this method is mainly used for temporary restorations, templates, and splints in recent years.

Reportedly, the most used chairside CAD/CAM ceramics of permanent restoration are silicate ceramics, and oxide ceramics, which show high mechanical resistance and excellent translucency. The high hardness, low elastic and plastic deformability and overall brittle behavior of these traditional ceramic materials also demonstrate low tolerance of deformation and abrasive wear on antagonist teeth as well [8]. In this sense, CAD/CAM hybrid materials, such as polymer-infiltrated ceramic, or resin-matrix materials with high ceramic content have been developed in recent years, which aim to combine the advantageous mechanical strength of ceramics and the flexibility of resin [6, 9]. Replacing the glass matrix of conventional ceramics with a polymer matrix, CAD/CAM hybrid materials are more tolerant to cracks and show similar mechanical performance to enamel and dentin and therefore are closer to natural materials and less abrasive towards these [10, 11]. Furthermore, with the continuous development of light-curing and 3D-printing technology, it is now possible to 3D-print CAD/CAM hybrid material for permanent single-tooth restorations [12].

In order to investigate and compare the properties of CAD/CAM hybrid permanent materials for milling and 3D-printing, in vitro tests such as mechanical strength or bonding tests are suitable [6, 13]. Several studies demonstrated superior mechanical properties of milled CAD/CAM hybrid material compared to 3D-printed ones [12, 14]. However, although bonding strength is of high clinical interest for the long-term stability of the restorations [6, 15, 16], compared evidence of bond strength between two CAD/CAM hybrid materials is currently rare. Moreover, except for material type differences, surface treatments may also play a significant role in strengthening the bonding interface by creating a micromechanical locking and chemical connection. For resin composite materials, etching or sandblasting followed by silane application was recommended [17, 18]. For hybrid ceramics, silane application after sandblasting with Aluminum oxide (Al_2_O_3_) or hydrofluoric acid etching was also shown to be beneficial [18, 19]. In general, a combination of mechanical (sandblasting) and chemical (etching, silanes) pretreatments could significantly increase the bonding strength [6, 17, 18, 20, 21]. Since new CAD/CAM hybrid materials have recently appeared on the market, suitable pretreatment measurements have not yet been conclusively clarified. In particular, data on 3D-printable materials and how they compare to millable materials are scarce.

The SBS test is one of the most widely used methods for testing the bond strength of a material [6, 22, 23]. Moreover, it is straightforward and highly reproducible to perform [6, 22, 23]. In addition, since the roughness and wettability of CAD/CAM material surfaces were found to have an effect on bond performance from previous studies groups [24, 25], investigating the changes of Ra and SFE following different treatments, is necessary to give a clear clue for the function of each pretreatment on bond strength of various materials. Strasser et al. argued that after sandblasting, high Ra and high SFE values were achieved on CAD/CAM hybrid materials [18]. Nevertheless, subjecting the CAD/CAM material to hydrofluoric etching for 60 s led to an average roughness increase (Ra, Rz values) of approximately 125%, which is combined with a decrease in SFE. Unfortunately, only one study compared the SBS value between 3D-printed and milled definitive restorations and argued that the 3D-printable material showed a lower SBS value than that of millable materials [4]. However, the influence of different conditioning strategies on bond strength, roughness, and surface energy has not been clarified.

Thus, the present study aimed to test the bonding performance of three different CAD/CAM hybrid materials for permanent restoration, two for milling and one for 3D-printing, after different pretreatments. The null hypotheses are that the 3D-printed (additively manufactured) CAD/CAM hybrid material presents no significant differences in bond strength compared to subtractively manufactured CAD/CAM hybrid materials and that pretreatment strategies would not influence Ra, SFE, and SBS of both materials.

## Materials and methods

### Specimen preparation

**Fig. 1:**
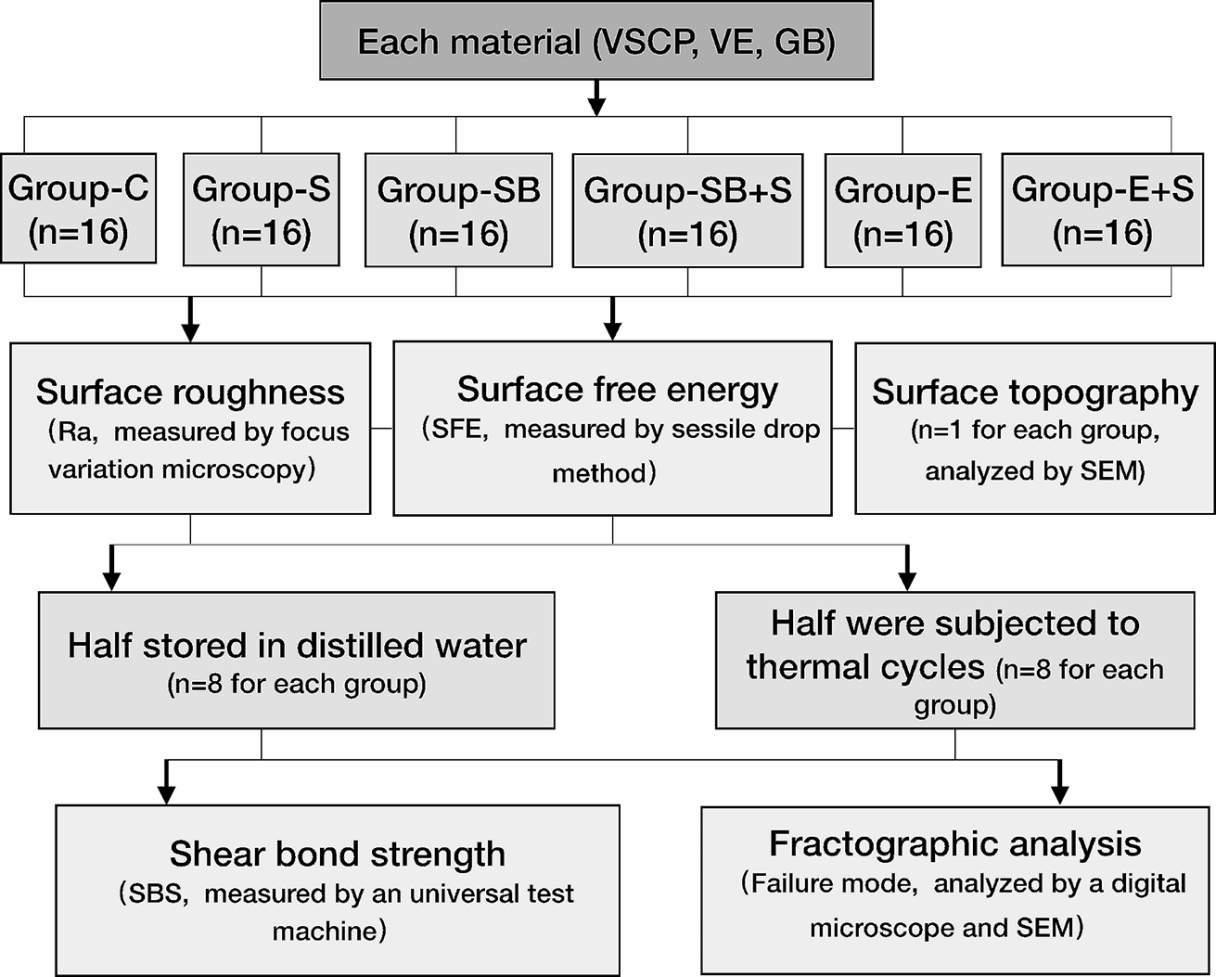
Flowchart presenting group assignment for each material

The details of three different CAD/CAM hybrid materials, two for milling and one for 3D-printing were described in Table 1. Rectangular specimens (20 × 10 × 2 mm) were designed in a CAD software (Fusion 360 CAD software; Autodesk, Mill Valley, CA, USA). Afterwards, the design files were exported to the standard tessellation language (STL) format. A total of 306 specimens (*n* = 102 for each material) were manufactured. The group assignment is shown in Fig. 1.

**Table 1:** The tested CAD/CAM hybrid materials of this study

Material	Composition	Manufacturer	Code
VarseoSmile Crown plus	Ceramic-filled (30–50 wt% inorganic fillers; particle size 0.7 μm) silanized dental glass, methyl benzoylfor-mate, diphenyl (2 ,4, 6-trimethylbenzoyl) phosphine oxide hybrid material	BEGO, Bremen, Germany	VSCP
Vita Enamic	Polymer infiltrated (UDMA, TEGDMA 14 wt%) feldspar ceramic network (86 wt%)	Vita Zahnfabrik, Bad Sackingen, Germany	VE
Grandio Blocs	Resin nanohybrid composite (86 wt% inorganic fillers), embedded in a polymer matrix (14% UDMA + DMA)	VOCO, Cuxhaven, Germany	GB

Source: [12, 26]

### Preparation of the specimens

The specimens of VSCP were fabricated by digital light processing (DLP) using a 3D printer (Varseo XS; BEGO, Bremen, Germany). All 3D-printed specimens were printed at an angle of 90 degrees on the build platform and with a layer thickness of 50 μm. The bottom of the specimens was attached to the support structure. After the printing process and the support structure removal, pre-cleaning and main-cleaning processes were performed. For this, the specimens were washed in a reusable 96% ethanol solution for 3 min in an unheated ultrasonic bath (Ultrasonic Cleaner; VWR, Darmstadt, Germany) to remove the remaining monomer content. Subsequently, all samples were removed from the ethanol bath and sprayed with 96% additional ethanol and dried with compressed oil-free air. Then the surface of the specimens was sandblasted with Perlablast micro (50 μm, BEGO) at a pressure of 1.5 bar to remove debonded ceramic particles. At last, all specimens were cured by a light curing machine (Otoflash, BEGO) with 1500 flashes on each side according to the manufacturer´s instructions. For the millable materials VE and GB, specimens were produced from corresponding CAD/CAM blocks using a 5-axis milling machine (vhf K5; vhf camfacture AG, Ammerbuch, Germany). After milling they were cut from the blocks by a universal cutter (U060-R2-40; vhf camfacture AG, Ammerbuch, Germany). For obtaining a standard surface morphology, the bonding surface of each specimen was grounded and polished by using 320-, 500- 800-, and 1200-grit silicon carbide papers (Hermes Schleifmittel GmbH, Hamburg, Germany) under constant water cooling for 30 s. Afterward, all specimens were ultrasonically cleaned in distilled water for 5 min to exclude surface contamination and dried using oil-free air immediately.

### Pretreatment of the specimens

After the manufacturing of all specimens, each material was randomly divided into six groups (*n* = 17) according to different pretreatment strategies:

- Control - C: No surface treatment.
- Silane - S: Silane (Monobond Plus; Ivoclar, Schaan, Liechtenstein) was applied using an applicator brush and dried for 60 s. The remaining silane was removed by compressed air for 5 s.
- Sandblasting - SB: The surfaces were subjected to air-abrasion with 50 μm aluminum oxide particles with a blasting device (Basic quattro; Renfert, Hilzingen, Germany) from a 10 mm distance at 2 bar propulsion pressure for 15 s. Compressed air was then applied to clean the surface.
- Sandblasting + Silane – SB + S: The above-described procedure of groups SB and S were combined. Sandblasting was performed before Silane was applied.
- Etching - E: The specimens were etched with 9% hydrofluoric acid (HF, Ultradent Porcelain Etch, Cologne, Germany) for 60 s and rinsed with distilled water for one minute. The surface was then air-dried.
- Etching + Silane – E + S: The pretreatment strategies of groups S and E were combined as described above. Etching was performed before the silane was applied.

### Microstructure and surface roughness (Ra value) analysis

To observe the surface topography, one specimen from each pretreatment group was selected and evaluated randomly with scanning electron microscopy (SEM) (Phenom XL; Thermofisher, Eindhoven, Netherlands) at 10,000 × magnification, without prior coating in low vacuum to prevent charging and with back scattered detector (BSD).

The Ra value of each specimen, representing the average of height deviations from the mean line, was measured using an optical roughness measurement instrument (Alicona InfiniteFocus G4; Alicona imaging GmbH, Raaba/Graz, Austria). Surface roughness analysis utilized a Gauss filter with a cutoff wavelength (λc) of 800 μm, and a focus variation microscope equipped with a 20X lens. The Ra value was determined over a profile length of 4 mm. The result of each specimen was averaged by three times equidistant parallel measurements. All measurements were recorded by one operator (Z.M).

### Measurement of surface free energy

The contact angle (CA) between the surface of the materials and different liquids was obtained on 3 specimens of each group by sessile drop technique with a digital microscope (KEYENCE VHX-500, Deutschland GmbH, Neu-Isenburg, Germany). The measurements on each specimen were carried out at room temperature with three liquids with varying and known dispersive and polar contents: distilled water, ethylene glycol, and glycerin. A 1-mL glass microsyringe was filled with the liquid and used to apply the drop on the sample surface. The process was operated manually by an experienced researcher (ZM). By pressing the piston, a 2µL droplet was formed at the tip of the needle and deposited on the sample’s surfaces. The contact angle was measured after 10 s wetting. To calculate SFE value, the mean contact angle values were used and calculations were performed in accordance with the model by Owens, Wendt, Rabel and Kaelble [27].

### Bonding of the luting material to the specimens

**Fig. 2:**
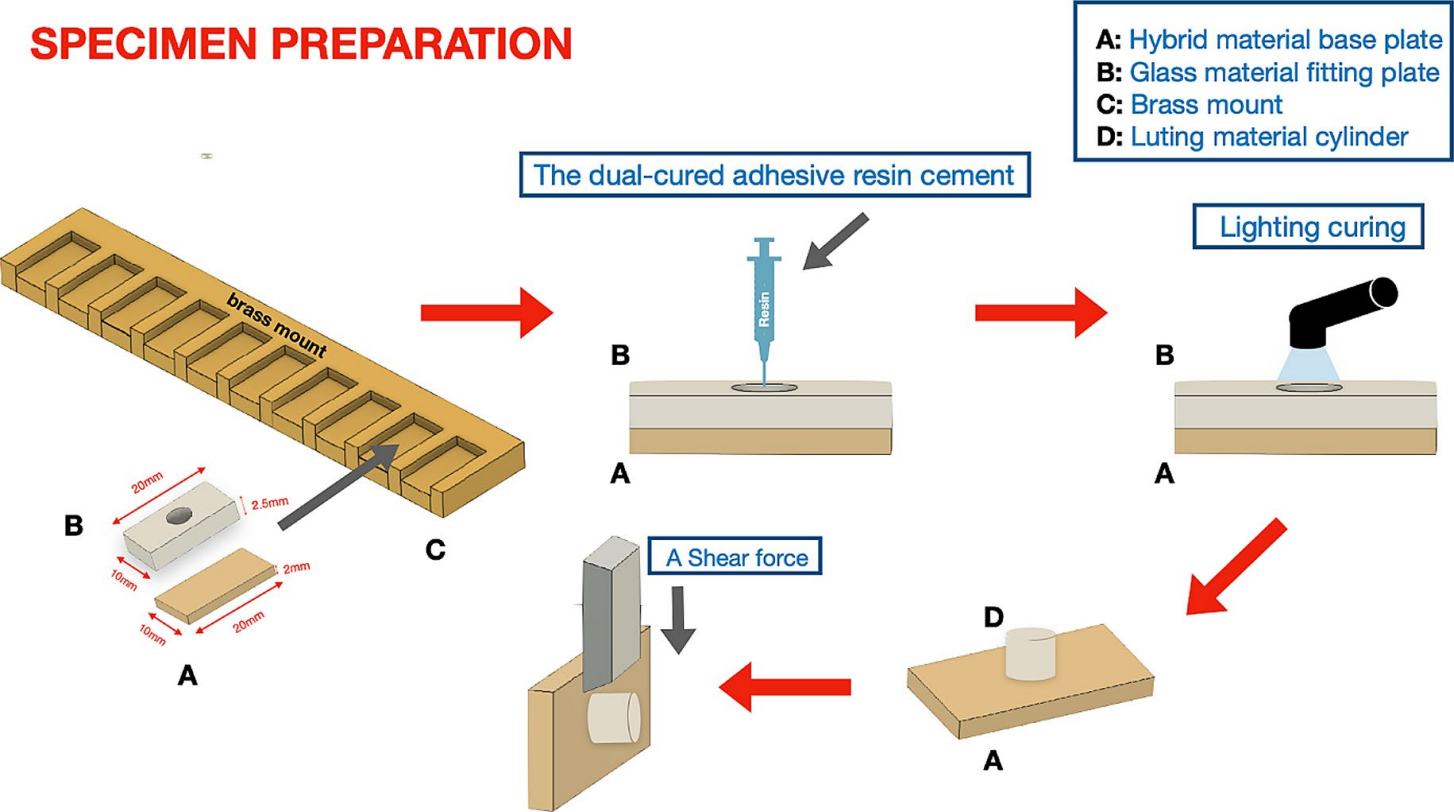
Flowchart of the specimen preparation. (A) CAD/CAM hybrid material base plate (A) was assembled in the slot of the brass mount (C). A fitting glass plate (A) was placed on top of the base material. Afterwards, a dual-cured adhesive resin material was applied to the cylindric hollow and a light polymerization of each layer was conducted for 20 s on each side. At last, the fitting plate was carefully removed and waited for the shear bond strength test

The flowchart of the bonding process is shown in Fig. 2. A brass mounting was fabricated to provide ten slots for the adhesive process. Each slot was filled with one specimen and a fitting plate (20 × 10 × 2.5 mm) with a 5 mm diameter central cylindric hollow pattern. A dual-cured adhesive resin cement (RelyX Ultimate; *Solventum*) was condensed to the central area in 1 mm incremental layers. Subsequently, light polymerization (385–515 nm intensity) (VALO Cordless; Ultradent Products, Cologne, Germany) of each layer was conducted for 20 s on each side. Afterward, the fitting plate was carefully moved by the operator (Z.M.).

### Water storage and thermal cycling

Half of the specimens of each pretreatment group (*n* = 8) were selected randomly for artificial aging. According to the International Organization for Standardization (ISO) standard 10,477 half of the samples of each group were stored in 37 °C distilled water for 24 h, while the remaining samples were subjected to 5000 thermal cycles in 5 and 55 °C water baths with 30 s of dwell time in each water bath and 5 s of transfer time.

### Shear bond strength test

The shear bond test was performed accordance with the DIN EN ISO 10477 standard for crown material in Dentistry [28]. SBS value was measured using a universal testing machine (Z010 Zwick/Roell, Ulm, Germany). A shear force was conducted with a cross head speed of 0.5 mm/min from the parallel direction of the bonding surface until fracture occurred. The failure load was recorded. Then SBS values of each specimen were calculated in megapascal (MPa) by dividing the fracture load (F) in Newton by the bonding surface area (A) in mm^2^:

1 $$SBS = \frac{F}{A} = \left[ {\frac{N}{{m{m^2}}}} \right]$$

### Fractographic analysis

After debonding, fractured interfaces were examined by a digital microscope (KEYENCE VHX-500, Deutschland GmbH, Neu-Isenburg, Germany) at a 30× magnification to determine the failure mode. Therefore, the following classification of failure modes was evaluated [29]: (1) adhesive failure in the bonding area; (2) cohesive failure in the inner part of adhesive luting material or composite resin itself; (3) Mixed failure consisting of both cohesive and adhesive failure. One specimen from each group was inspected randomly via scanning electron microscopy at 1000 x magnification to observe the fractured surface topography.

### Statistical analysis

The mean and standard deviation (SD) of values in each group were statistically analyzed by using GraphPad Prism 9.0 (GraphPad Software, Boston, MA, USA) software. The influence of two variables (composite material types, surface treatment method) on the Ra values, SFE values and SBS values was assessed by two-way analysis of variance (2-Way ANOVA). The Shapiro-Wilk test (*p* > 0.05) was performed to determine the normality of data distribution. The independent t-test and paired-sample were Mann-Whitney-U-test performed to investigate the influence of artificial aging of each material. The statistical difference of frequencies of failure mode was analyzed by the chi-square test. Statistical significance was set as *p* < 0.05 probability level.

## Results

### Surface roughness

The mean Ra values were significantly affected by material type (*p* = 0.001), conditioning strategy (*p* < 0.001), and material type × conditioning strategy interaction (*p* < 0.001). The average Ra of the bonding interface following surface conditioning is provided in Table 2. Among all treatment methods, sandblasting with/without silane applied significantly increased Ra in all material groups. Etching with/without silane applied only improves Ra significantly in VE. The highest values were identified in VSCP specimens with treatments of sandblasting plus silane strategy, while the lowest values were represented in GB specimens without pretreatment.

**Table 2:** Mean ± standard deviation (SD) of surface roughness (Ra) for each group according to the surface treatment and CAD/CAM hybrid material types (µm)

	VarseoSmile Crown ^plus^	Vita Enamic	Grandio blocs
**Surface treatment**	**Mean/SD**	**Mean/SD**	**Mean/SD**
C	0.24 ± 0.07	0.24 ± 0.12	0.22 ± 0.08
S	0.36 ± 0.10	0.42 ± 0.12	0.39 ± 0.11
SB	3.23 ± 0.54****	3.05 ± 0.63****	3.17 ± 0.45****
SB + S	3.75 ± 0.72****	3.05 ± 0.40****	3.07 ± 0.45****
E	0.34 ± 0.13	0.98 ± 0.19****	0.29 ± 0.10
E + S	0.45 ± 0.19	1.03 ± 0.21****	0.50 ± 0.30

C, control; S, silane; SB, sandblasting; E, Etching; When compared with the control group, *indicate *p* < 0.05, **indicate *p* < 0.01,****indicated *p* < 0.001

**Fig. 3:**
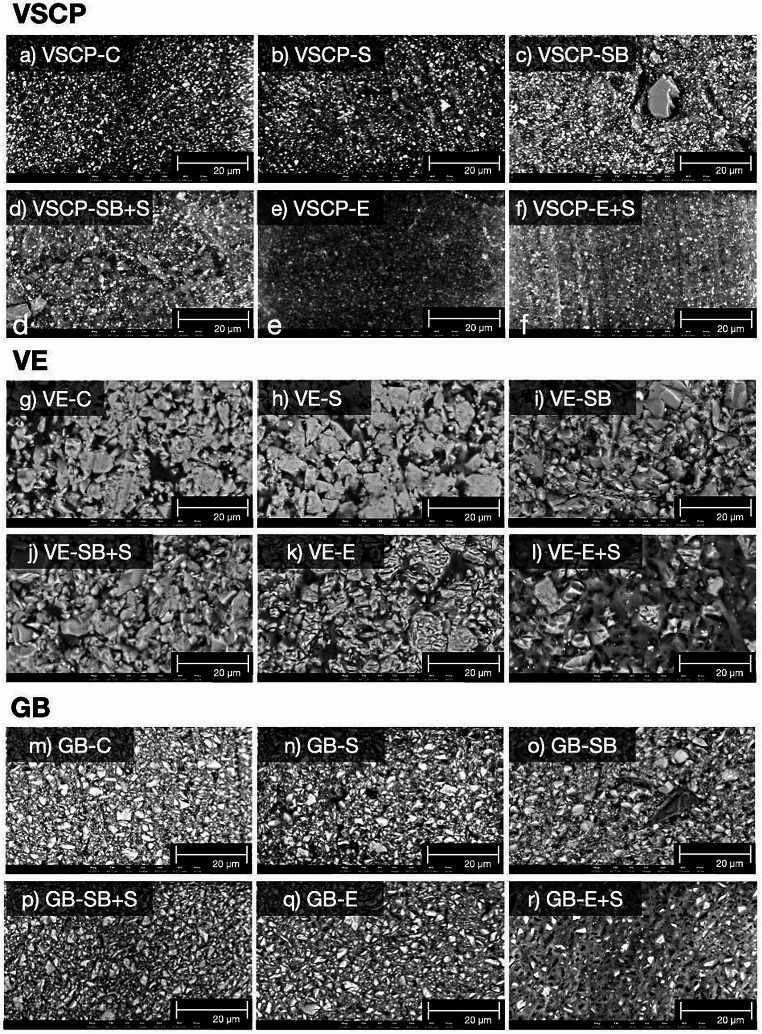
SEM images of surfaces of three test specimens after surface treatments (magnification × 10.000). VarseoSmile Crown plus: a no treatment; b silane; c sandblasting; d sandblasting + silane; e acid etching; f acid etching + silane; Vita Enamic: g no treatment; h silane; i sandblasting; j sandblasting + silane; k acid etching; l acid etching + silane; Grandio blocks: m no treatment; n silane; o sandblasting; p sandblasting + silane; q acid etching; r acid etching + silane

The surfaces of one sample per treatment for each material (VSCP, VE, and GB) were analyzed using SEM imaging with backscattered detector, which will give elemental contrast, therefor allowing to distinguish between polymer (dark gray) and ceramic (light gray) phase (Fig. 3). The images from the non-treated groups revealed differences regarding the ceramic and resin contents and the particle diameter among the three tested materials. The shape of the ceramic particles was similarly irregular in all materials. VSCP (Fig. 3a) showed the smallest filler particles and the lowest filler content; while VE (Fig. 3g) showed a ceramic particle network consisting of larger particle size up to ~ 10 μm infiltrated with resin. GB (Fig. 3m) presented a finer surface structure compared to VE with particles up to around 5 μm and a high filler content. Meanwhile, sandblasting (VSCP: Fig. 3c, VE: Fig. 3i, GB: Fig. 3o) generated more irregularities on the surface than any other pretreatment method. The application of acid resulted in deeper irregularities on VE and GB surfaces. In VE it appears that the ceramic particles surface became rougher after etching, in GB the change in surface is much less visible. Due to the smaller ceramic particle size in GB it appears that more ceramic particles in the surface were dissolved, leaving behind more polymer resin. However, the influence of etching on the surface of VSCP specimens appeared heterogeneously.

### Surface free energy

The mean SFE values were significantly affected by pretreatment strategy (*p* < 0.001), and material type × pretreatment strategy interaction (*p* < 0.001), but not by material type (*p* = 0.406). The average SFE of the bonding interface following surface conditioning is provided in Table 3. There were no significant differences in SFE with or without most surface treatments applied (*p* > 0.05), while only etching on VSCP and VE surface increased SFE values significantly.

**Table 3:** Mean ± standard deviation (SD) of surface free energy (SFE) for each group according to the surface treatment and CAD/CAM hybrid material types (mJ/m^2^)

	VarseoSmile Crown plus	Vita Enamic	Grandio blocs
**Surface treatment**	**Mean/SD**	**Mean/SD**	**Mean/SD**
C	29.28 ± 7.33	31.71 ± 1.73	35.98 ± 4.05
S	29.86 ± 1.38	48.94 ± 11.94	34.63 ± 3.31
SB	26.70 ± 5.94	35.87 ± 5.54	58.28 ± 13.41
SB + S	32.85 ± 11.78	33.76 ± 14.65	33.11 ± 2.72
E	59.22 ± 11.79*	61.13 ± 8.93*	44.29 ± 3.99
E + S	49.42 ± 7.85	36.59 ± 3.57	41.32 ± 6.29

C, control; S, silane; SB, sandblasting; E, Etching; When compared with the control group, *indicate *p* < 0.05

### Shear bond strength

**Fig. 4:**
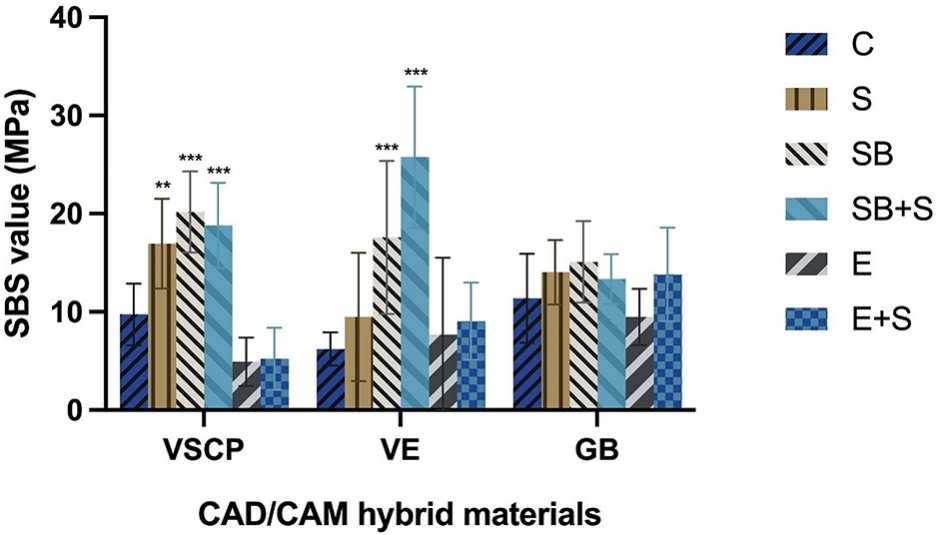
The SBS value of different surface treatments on the three tested materials. C: control, no surface treatment; S: silane; SB: sandblasting; SB + S: sandblasting + silane; E: acid etching; E + S: acid etching + silane: when compared with the SBS values in control group, * differences are significant at *p* < 0.05, ** differences are significant at *p* < 0.01, and *** differences are significant at *p* < 0.001

The mean SBS values were statistically significantly influenced by the surface pretreatment strategy (*p* < 0.001) and interaction (*p* < 0.001), except for the material type (*p* = 0.937). It was observed that VE specimens treated with sandblasting and silane application in combination resulted in the highest SBS values, while the etching group of VSCP specimens showed the lowest mean values (Fig. 4). Regarding pretreatment strategies, etching (*p* = 0.242), also in combination with silane (*p* = 0.171), did not show significant effects on SBS in all materials. Besides, the use of silane alone only significantly increased SBS in VSCP (*p* < 0.05), while sandblasting with or without silane applied, all improved SBS significantly in VSCP and VE (*p* < 0.01). In contrast, no significant influence was found between all pretreatment groups and the control group in GB.

### Thermocycling

**Fig. 5:**
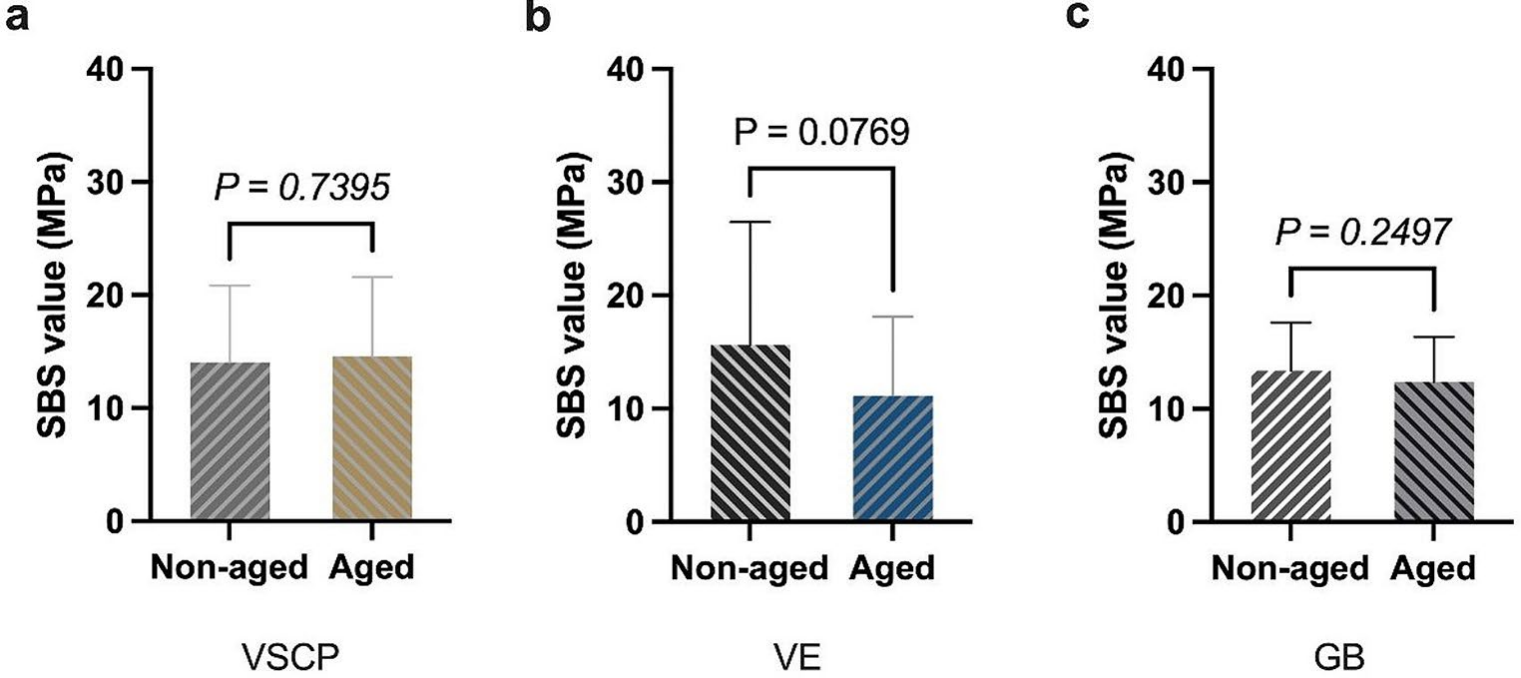
The SBS value of the tested materials with or without thermal cycling; No significant difference was found between the two aging conditions for the three tested materials

The direct comparison between the aged and non-aged groups of all tested materials (VSCP, *p* = 0.740; VE, *p* = 0.077; GB, *p* = 0.250) revealed that there was no significant difference regarding SBS values before and after thermocycling (Fig. 5).

### Fractographic analysis

**Fig. 6:**
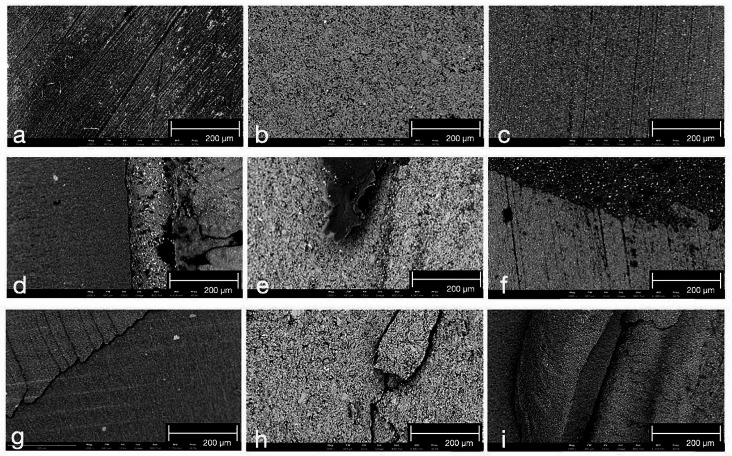
SEM images of the fracture surface of the failure mode (magnification × 1,000): a-c (adhesive failure), a VarseoSmile Crown plus; b Vita Enamic; c Grandio blocs; d-f (mixed failure), d VarseoSmile Crown plus; e Vita Enamic; f Grandio blocs; g-i (cohesive failure), g VarseoSmile Crown plus; h Vita Enamic; i Grandio blocs

Chi-square analysis indicates that material type (*p* < 0.05) and surface treatment methods (*p* < 0.05) significantly influenced the distribution of failure modes, while no significant difference was established in aging conditions (*p* > 0.05). For all materials, the adhesive failure occurred mostly in the control and etching groups, while sandblasted and silane-treated specimens showed predominantly cohesive and mixed failure types (Table 4). It was shown that around half of the VSCP specimens (53.2%) and VE specimens (49.4%) showed cohesive failure, whereas adhesive failure dominated most in GB specimens (40.6%). Three different failure modes of each material were shown by SEM imaging (Fig. 6) after the SBS tests. The residual resin luting agent was found in mixed failure modes while microcracks within the composite material were observed in cohesive failure modes.

**Table 4:** Number of failure modes after SBS tests

Material	Pretreatment (group)	Adhesive failure (*n*)	Mixed failure (*n*)	Cohesive failure (*n*)
VarseoSmile Crown plus	C	7	7	2
	S	0	4	12
	SB	0	4	12
	SB + S	0	0	15
	E	7	1	0
	E + S	5	1	0
Vita Enamic	C	14	1	0
	S	5	4	5
	SB	0	0	15
	SB + S	0	0	16
	E	6	0	1
	E + S	6	4	2
Grandio blocs	C	16	0	0
	S	1	11	4
	SB	3	7	6
	SB + S	2	8	6
	E	14	2	0
	E + S	3	9	4

C, control; S, silane; SB, sandblasting; E, Etching

## Discussion

In the present study, the tested CAD/CAM hybrid materials reacted differently to pretreatments. The first null hypothesis can be accepted since no significant difference regarding shear bond strength was found among the three tested materials. The second null hypothesis must be rejected because various pretreatments showed a significant influence on the bond properties of each material.

Before applying resin cement, certain pre-treatment procedures on the surface may play a vital role in obtaining micromechanical retention. In general, it could be proven that a higher roughness of the surface caused by mechanical pretreatment leads to a higher bond strength compared to chemical pretreatment [17, 18]. This conclusion could also be shown in the present study. Due to the generation of microretentive ridge and groove patterns by sandblasting, Ra is increased. The highest Ra values of all tested materials were obtained after pretreatment by sandblasting or sandblasting + silane application, which showed the highest SBS values at the same time. These findings were consistent with a previous study, which argued that a recommendation of airborne-particle abrasion appears reasonable for VSCP [30]. On the one hand, the present study showed that hydrofluoric etching presented the higher SFE values. On the other hand, it showed lower bond strength than the mechanically pretreated groups. This phenomenon may be due to the fact that SFE is a fundamental material property that is influenced by the inherent chemical composition of the material rather than surface roughness. In contrast, improving surface roughness, e.g. by sandblasting, may lead to higher wettability and therefore to a lower contact angle by increasing the effective surface area which is available for liquid interaction, therefor it may influence the measured SFE values, even though it may not change the composition of the surface. Acid etching typically results in an increase in functional groups such as hydroxyl groups on the surface, which enhance the interaction between the surface and liquids. Consequently, the surface exhibits a higher SFE. However, the resistance of the polymer matrix to acid etching might be influenced by type of polymer, acid concentration or durations. Therefore, the function of etching might be various in different surfaces. It is noticeable that the application of acid resulted in deeper irregularities on VE surfaces (Fig. 3k and g) compared to VSCP (Fig. 3a and e). Similarly, the lowest bond strength in the group of etching was shown in VSCP, which has the highest resin polymer content of all three investigated materials. Thus, it seems that the function of etching on the polymer matrix is not as effective as sandblasting to achieve micro retention, even though it led to an increase in SFE in all materials. It follows that increasing roughness is more essential for achieving adequate retention on CAD/CAM hybrid material surfaces than altering surface free energy. Similarly, Park et al. demonstrated that hydrofluoric etching on the surface of CAD/CAM hybrid materials resulted in lower roughness and tensile bond strength compared to air abrasion [31]. In summary, the evidence is clear that mechanical pretreatment is crucial for CAD/CAM hybrid materials to obtain higher bond strength regardless if they are millable or 3D-printable ones.

Regarding millable CAD/CAM hybrid materials, the highest bond strength for VE was observed in Group SB + S, while GB reached the highest SBS value when sandblasting without silane was applied. That may be due to the difference in composite structure. Different structures of two materials were found in Fig. 3g and m. GB is considered as a dispersed filler structure, since the inorganic micron and submicron sized fillers are dispersed within the resin matrix [12]. In contrast, VE is composed of an open porous feldspathic ceramic structure network, which is infiltrated with an acrylate cross-linked polymer network [32]. Both networks can chemically link to the methoxy groups of the silane [33]. According to the results, Ra values of Group SB and Group SB + S are similar in VE while significantly higher bond strength can be found in the latter group. This might be a result of surface inherent chemical composition alteration caused by silane. In fact, the silanization process may decrease the polar fraction while concurrently increasing the dispersive fraction in the same proportion. This phenomenon may lead to notable alterations in surface hydrophilicity and bond strength, while the SFE values remain unaltered following silane application. In addition, it should not be ignored that the SFE values and SBS values remained stable on the GB surface regardless of any pretreatment applied. This demonstrated that the mentioned surface treatments cannot provide sufficient contribution for surface activation in the dispersed filler structure of GB. This phenomenon was also reported by Günal-Abduljalil et al. who argued that silane application only increased the SBS values of the VE rather than GB [29]. The findings of the present study revealed that silane application should not be avoided after mechanical treatment on VE surface, whereas the strategies selection for GB can be less strict.

The surface of 3D-printable CAD/CAM hybrid materials may react differently to treatments compared to millable ones. According to ISO standard 10,477, bond strength for composite system should not be less than 5 MPa to maintain an acceptable retention. In the present study, all test materials demonstrated sufficient bond strength with or without surface treatment, except for the VSCP etching group, which presented significantly lower SBS value (4.92 ± 2.47 MPa). This may be due to the lower inorganic filler and smaller filler sizes of the 3D-printed material. In general, by partial dissolution of the glassy phases of the ceramic, hydrofluoric etching surface treatment modifies the microstructures of the ceramic inorganic surface and increases the surface area. This may be beneficial to the mechanical interlocking with the adhesive resin. It has been reported that acid etching is considered as the golden standard for glass ceramics, whereas the effect of etching on CAD/CAM hybrid materials remains unclear due to the lower inorganic filler content. A previous study demonstrated that hydrofluoric etching may achieve better bond strength in glass ceramics rather than hybrid materials [34]. Cekic-Nagas et al. found that hydrofluoric acid gel treatment had no effect on bond strength values on millable CAD/CAM hybrid materials [15]. As low inorganic filler content cannot be avoided to acquire flowable consistency during 3D-printing, the hydrofluoric etching on the VSCP surface presented even lower bond strength than the milled CAD/CAM hybrid materials. Secondly, in terms of silane application, despite increased SBS values observed in all tested materials, significant improvement was only found in the VSCP surfaces. This may relate to the inorganic content ratio and SFE value. It is generally accepted that hydroxyl groups and bifunctional monomer of silane are able to react with both the inorganic composition and polymer content groups respectively. Meanwhile, by wetting the conditioned surface, the silane coupling increased micromechanical interlocking and accomplished the silanization process by chemical bonding on the surface of hybrid materials [35, 36]. Therefore, as the organo-functional group of the silane is assumed to link with the organic resin monomer, it is possible that with lower inorganic content and lower SFE values than milled materials, the VSCP surface could be activated by silanes to a greater extent. Furthermore, other than silane coupling agents, the function of 10-methacryloyloxydecyl dihydrogen phosphate (10-MDP) from Monobond plus may contribute to the results as well. The methacrylate functional group within 10-MDP may undergo radical polymerization alongside other methacrylate monomers, culminating in the formation of a polymer network on the material surface [37]. In other words, on a 3D-printing material that is characterized by higher organic content and the potential presence of residual monomers subsequent to the printing and curing process, the influence of 10-MDP may be more pronounced compared to milled materials. However, due to the lack of evidence, this hypothesis should be tested in more clinical and in vitro studies to compare the differences of silane application on 3D-printed and milled CAD/CAM hybrid materials.

Thermocycling did not show any effect on the SBS of all tested CAD/CAM hybrid materials. This is similar to the results of a previous study by Graf et al. [30]. According to other studies, thermocycling is used to influence retention negatively [38–40]. The difference may be influenced by various thermal cycles. Regarding the fractographic analysis, it could be shown that lower SBS values after chemical pretreatment led to adhesive failures whereas higher SBS values after micromechanical pretreatment led to cohesive and mixed failures. In summary, results from the present study indicated that there is no significant difference in SBS value between 3D-printed and millable CAD/CAM hybrid materials in general. However, to achieve better performance in shear bond strength, sandblasting and silane coupling can be recommended for all tested materials in general, whereas hydrofluoric etching is not suitable for CAD/CAM hybrid materials.

## Limitations

There are several limitations in the current study. Firstly, only one sandblasting and one etching strategy have been evaluated. Thus, the results can only be applied on limited conditions. Meanwhile, in vitro design is limited to fully replicate the real intraoral condition. In a clinical approach surfaces are not polished prior to luting, as we did in our study. Therefore the effect of all investigated surface treatments may vary in a clinical set-up. The evaluating methods in the current study for surface roughness and SFE values have its limitations. For instance, applying the drop manually by the observer and determining the contact angle subjectively may lead to variability in results. The polar and dispersive fractions values were also not investigated in detail. In addition, contact angles should ideally be investigated on polished surfaces, as wettability increases with increasing surface roughness, so the investigation of SFE on e.g. sandblasted surfaces may have given false values of SFE, due to the increased roughness. Moreover, although SBS test has been widely used to test in vitro studies to investigate dental ceramic materials, the uniform stress distribution across the material from the experiment may be a problem that cannot accurately represent the true bonding capacity of resin composites to ceramic surfaces in practical applications. Therefore, caution should be exercised when interpreting SBS test results. Replaced methods such as tensile bond strength test, or bond strength testing under simulated physiological conditions should also be recommended in future investigations. In order to be able to better classify these initial findings in a direct comparison of millable and printable CAD/CAM hybrid materials, further clinicals studies are desirable.

## Conclusions

With the limitations of this in vitro study, the following conclusions can be drawn:

1. Surface roughness exerts a greater impact on the bond strength of CAD/CAM hybrid materials when compared to surface energy.
2. Shear bond strength values between additively and subtractively manufactured CAD/CAM hybrid materials presented no significant difference.
3. Micromechanical pretreatment seems to be superior to chemical conditioning regarding bond strength for all tested CAD/CAM hybrid materials.
4. Al_2_O_3_ sandblasting and silane coupling were suitable for both millable and printable materials, while hydrofluoric etching should not be recommended for CAD/CAM hybrid ceramic materials.
5. Artificial aging within 5000 thermal cycles does not influence the shear bond strength value of the tested materials.

## Data Availability

The authors confirm that the data supporting the findings of this study are available within the paper. Should any raw data files be needed in another format they are available from the corresponding author upon reasonable request.
